# Examining the spatial variations of co-morbidity among young children in Ethiopia

**DOI:** 10.1186/s12887-020-02198-3

**Published:** 2020-06-19

**Authors:** Kasahun Takele, Temesgen Zewotir, Denis Ndanguza

**Affiliations:** 1 African Center of Excellence in Data Science, University of Rwanda, Kigali, Rwanda; 2 School of Mathematics, Statistics and Computer Sciences, University of KwaZulu-Natal, Durban, South Africa; 3 College of Science and Technology, University of Rwanda, Kigali, Rwanda

**Keywords:** Child, Cough, Diarrhea, Fever, Multinomial

## Abstract

**Background:**

Addressing the issues of childhood comorbidity remains a crucial global public health issue due to its consequences in child wellbeing. This study aims to account for nonlinear, spatial effect and to evaluate spatial variation in childhood co-morbidity at cluster level while controlling for important risk factors.

**Methods:**

Using the 2016 Ethiopia DHS data, a multinomial logistic model was assessed by linear, nonlinear and random effects. The study also employed a spatial analysis tool which is Getis-Ord to identify hotspot areas of child comorbidity at the cluster level. The model with fixed, nonlinear and spatial effects identified as the best model to identify risk factors related to the coexistence of childhood illnesses.

**Results:**

The results indicated that statistically significant high hotspots of comorbidity were found in Tigray and Oromia whereas low hotspots were found in Harari and Somali regions. Children between 10 and 15 months old were at high risk of co-morbidity in Ethiopia. Besides, our findings revealed that being male children, not-breastfed children, from households lack of toilet facility, children from households who use spring water, children born first, children from working mother, anemic children and children from uneducated mother are at high risk of multiple illnesses.

**Conclusions:**

Comorbidity in childhood is not random in the country, with high hotspots of comorbidity in the regions of Tigray and Oromia. The results show a critical upshot for a combined morbidity control method for decreasing children’s illnesses and death. The maps remain novel to design appropriate healthcare interventions at regional as well as cluster level. Regions with high hotspots of child comorbidity should be considered for health healthcare interventions.

## Background

Addressing the issues of childhood morbidity is a priority for the public health agenda since it remains the burden of child wellbeing specifically in developing countries. In sub-Saharan Africa, the foremost causes of childhood morbidity and mortality are diarrhea, cough and fever [1]. In spite of improvements in fighting childhood illnesses, communicable diseases remain a foremost reason of death for children. For instance, diarrhea (8%), pneumonia (15%) and malaria (5%) account for global children’s death. In 2018, in Sub-Saharan Africa, one in thirteen children dies before his/her fifth birthday, which is sixteen times higher than the average ratio of one in one hundred ninety-nine in high-income countries [2]. Illnesses are unlikely to be uniformly distributed in a population, having income and regional disparities. Moreover, children might experience compound illnesses simultaneously or repeatedly, increasing the risk of severe disease or death [3].

Likewise, in Ethiopia, children’s illness and death persist high because of the problem related to predominant sicknesses such as diarrhea, malaria, fever and cough. In 2016, 12% of under-age of five children had a diarrheal incident, 14% had a fever incident and 16% had a cough incident in the 2 weeks before the survey. Diarrhea contributes to 1 in 10 (13%) child deaths in Ethiopia [4].This specifies that addressing the issue of childhood illness plays a fundamental role in addressing the issue of under-5 child mortality circuitously.

Furthermore, spatial location is a proxy of socioeconomic and environmental risk factors that affect childhood morbidity prevalence. For that reason, determinations to decrease the problem of childhood morbidities should examine the influence of spatial effect on child well-being. Some of the literature on childhood morbidities acknowledges as it has variation and varied geographically [5–7]. For instance, a study conducted in Egypt by [5] revealed that childhood morbidity has a geographically clustered pattern. In the previous study on diarrhea, unsafe water, wasting and unsafe sanitation were the foremost risk factors for diarrhea, accountable for 80·4% of diarrhea deaths in children below-5 years [8]. Furthermore, thus far, few studies in developing countries have examined the relations among child’s health, spatial variation, demographic, socio-economic and the environment in the distinct societies for a particular disease at a time [6, 7, 9–12]. However, the disease outcomes (diarrhea, fever and cough) frequently coexist and may share intersecting risk factors. Consequently, segregated analyses possibly will fail to give a complete depiction of the epidemiology of the comorbidities at the population level. In addition, some of the risk factors like age of child and spatial effect may not have a linear effect on the coexistence of childhood morbidities.

On the other hand, no previous study has used a method for analyzing nonlinear and spatial effects related to the coexistence of childhood diseases in Ethiopia using the Bayesian framework. The extent to which nonlinear and spatial affects the comorbidities remains poorly understood, also little has been known about the multiple illnesses. Considering the coexistence of diarrhea, fever and cough are critical for policymakers to understand the occurrence of illnesses to design for appropriate healthcare interventions at the community level. Therefore, in this paper, we aim to apply the Bayesian multinomial logistic model to account for nonlinear, spatial effect and to evaluate spatial variation in childhood comorbidities of diarrhea, fever and cough while controlling for important risk factors.

## Methods

### Data source

**Fig. 1:**
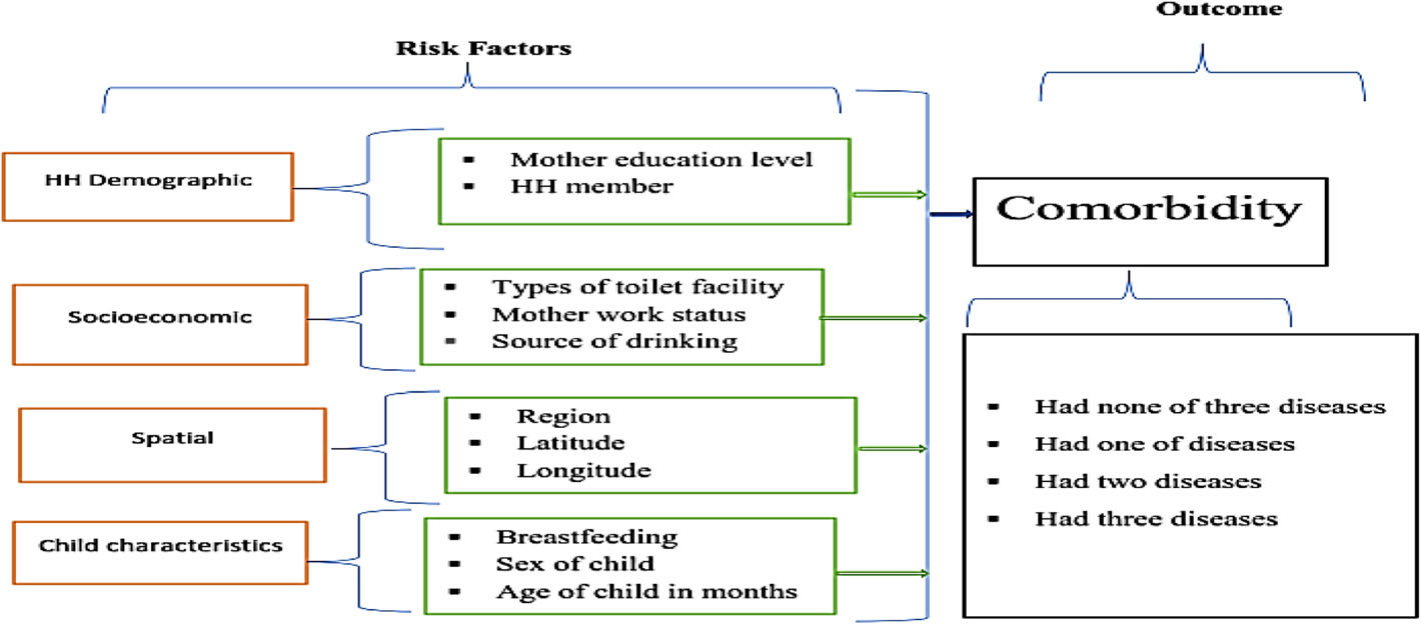
Selected risk factors of childhood comorbidity

We used data from the 2016 Ethiopia DHS. The survey has collected data on childhood and mother wellbeing conditions in the country every 5 years using 2-stage sampling, stratified by state and urban-rural areas. It provides national and regional level estimates of children and population health indicators. All childbearing age women (15–49 years) were suitable for interviews. Questions employed to determine current occurrences of the three morbidities were: “Does/Did the child had diarrhea/ fever/cough in the last two weeks?” Generally, 8742 data for diarrhea, fever and cough were collected [4]. Then the incidence of diarrhea/fever/cough considered as “Yes” or “No.” Risk factors considered in the study were child’s sex, child’s age, region, breastfeeding, types of toilet facility, place of delivery, mother’s work status, household size, mother’s educational level, childbirth order and source of drinking water. Each observation linked to 11 regions, as the data were geo-referenced and spatial analysis is reasonable. Selected risk factors of childhood comorbidity are shown in Fig. 1.

### Statistical methods

When studying childhood diseases, the intention is to associate the response variable “comorbidity” to socioeconomic, demographic, health and spatial covariates. Henceforth, from a statistical viewpoint, the outcome variable is given by a nominal categorical variable (*y*_*ijl*_) with unranked categories. Where *y*_*ijl*_ be the sickness status and *π*_*ijl*_ be the probability of multiple comorbidity of child *j*, *j* = 1, …, *n*_*i*_ in region *i*, *i* = 1, …, 11. Then the comorbidity of a child is coded as:

$$ {y}_{ijl}=\left\{\begin{array}{l}l= 0\kern0.48em had\kern0.24em non\kern0.34em of\kern0.34em illnesses\\ {}l= 1\kern0.48em had\kern0.24em one\kern0.24em illness\\ {}l= 2\kern0.48em had\kern0.24em two\kern0.24em illness es\\ {}l= 3\kern0.36em had\kern0.24em three\kern0.34em illnesses\end{array}\right. $$

Then, the most protuberant logistic model for this condition is the multinomial logit model [9, 13]. Consequently, the distribution of *y*_*ijl*_ is a multinomial i.e. *y*_*ijl*_~*M*(1, *π*_*ijl*_), and *π*_*ijl*_ = (*π*_*ij*0_, *π*_*ij*1_, …, *π*_*ij*3)_. Given some categorical risk factors, *H*_*ij*_, continuous variables, *U*_*ij*_ and region specific random effect, *S*_*il*_, the likelihood of co-morbidity can be modeled as:

1 $$ {\pi}_{ijl}=\frac{\mathit{\exp}\left({\eta}_{ijl}\right)}{1+\sum \limits_{i=1}^l\mathit{\exp}\left({\eta}_{ijl}\right)},l=0,1,\dots, 3 $$

Where

2 $$ {\eta}_{ij l}={H}_{ij}{\beta}_l+{f}_l\left({U}_{ij}\right)+{S}_{il} $$

is a predictor. *η*_*ijl*_ is a known response function with a logit link function, *β*_*l*_ is the vector of the regression parameters for each illness status *l*, *f*_*l*_ is a smooth function for the continuous risk factors and *S*_*il*_ are the spatial effects that further split up into a spatially structured (correlated) (*f*_*str*_) and an unstructured (uncorrelated) (*f*_*ustr*_) effects, that means, *S*_*il*_ = *f*_*str*_ + *f*_*ustr*_. We consider exp(*β*_*l*_) as a relative odds ratio for interpretation. To estimate model parameters we employed the fully Bayesian method. In a Bayesian setting, functions, linear parameters and the variances are considered to be random variables and appropriate prior distributions are assigned to them. For the fixed effect parameters, we assume independent diffuse priors, that is, *β*_*l*_ ∝ *const* in the absence of prior knowledge [13, 14]. Dispersed Gaussian priors also used as alternative. For the smooth functions *f*_*l*_, we used Bayesian version of P-spline, the prior suggested by Lang and Brezger [15]. Smooth functions are nonparametric function and the prior permits the function to be estimated as linear combinations of the basis function (B-spline). Thus, we obtain:

3 $$ f(x)=\sum \limits_{t=1}^m{\upbeta}_t{B}_t(x) $$

where *B*_*t*_(*x*) are basis functions and β_*t*_ are vector of unknown regression coefficients. Furthermore, the vector of unknown regression coefficients (β_*t*_) are elucidated to conform to a second-order random walk

4 $$ {\beta}_{(t)}=2{\beta}_{\left(t-1\right)}-{\beta}_{\left(t-2\right)}+{\epsilon}_{(t)} $$

with Gaussian errors *ϵ*_*t*_~*N*(0, *τ*^2^) and the diffuse priors *β*_(1)_ ∝ *const* and *β*_(2)_ ∝ *const*, for starting values. Where *τ*^2^ is used to control the smoothness of the *B*-spline function. Weakly informative inverse Gamma prior *τ*^2^~*IG*(*a*, *b*) were assigned to estimate the variance (*τ*^2^) jointly with the *B*-spline function.

For the spatial part, Markov random field priors are common in spatial statistics specifically for the spatially correlated effect *f*_*str*_ [9, 16, 17]. In addition, it indicates that the spatial neighborhood relationships provided that the two regions share a common boundary. Hence, a spatial extension of the random walk model provides the conditional spatially autoregressive condition [6, 18]. The spatial smoothness prior is denoted by

5 $$ {f}_{str}(s)\mid {f}_{str}(r),r\ne l\sim N\left(\sum \limits_{r\in {\phi}_l}{f}_{str}(r)|{N}_s,{\tau^2}_{str}|{N}_s\right) $$

where *N*_*s*_ is the number of neighborhood regions and *r* ∈ *ϕ*_*l*_ represents that region *r* is a neighbor of regions *s*.The conditional mean of *f*_*str*_(*r*) is the average function evaluations of neighboring regions. Besides, *τ*^2^_*str*_ used to control the amount of spatial smoothness. For a spatially unstructured effect *f*_*ustr*_, a common assumption is that the parameters *f*_*ustr*_ (*S*) are identically and independently distributed Gaussian. Then, it is denoted by

6 $$ \raisebox{1ex}{${f}_{unstr}(s)$}\!\left/ \!\raisebox{-1ex}{${\tau^2}_{unstr}$}\right.\sim N\left(0,{\tau^2}_{unstr}\right) $$

Flexibility and the degrees of smoothness of the trade-off is controlled by the variance parameter *τ*^2^, *str*, *unstr*. Furthermore, the inverse Gamma hyper-prior *τ*^2^~*IG*(*a*_*j*_, *b*_*j*_) is assigned to the variance *τ*^2^. Implies that for *τ*^2^_*str*_ and *τ*^2^_*unstr*_ we assume *τ*^2^_*str*_~*IG*(*a*_*str*_, *b*_*str*_) and *τ*^2^_*unstr*_~*IG*(*a*_*unstr*_, *b*_*unstr*_). Usual selections for the hyper-parameters are *a* = 1 and *b* = 0.005 or *a* = *b* = 0.001 [14]. Then, the probability density function expressed as

7 $$ p\left({\tau}^2\right)\propto {\left({\tau}^2\right)}^{-{a}_{j-1}}\exp \left(-{b}_j|{\tau}^2\right) $$

Bayesian inference is based on posterior distributions and is carried out using MCMC simulation techniques so that samples are drawn from full conditionals of single parameters or block parameters given the rest. Let *α* denote the vector of all unknown parameters in the model (i.e. *α* = (*f*, *f*_*spat*_) and *τ* represent the vector of all variance components. Then, under usual conditional independence assumptions, for the multinomial logit model, Bayesian inference can be based on the posterior given by

8 $$ p\left(\alpha |{y}_{ijl}\right)\propto \prod \limits_{i=1}^n{L}_i\left({y}_{ijl},{\eta}_{ijl}\right)\prod \limits_{j=1}^p\left\{p\left({\beta}_j|{\tau_j}^2\right)p\left({\tau_j}^2\right)\right\}p\left({f}_{str}|{\tau_{str}}^2\right)p\left({f}_{unstr}|{\tau_{ustr}}^2\right)\prod \limits_{j=1}^k\left({\gamma}_j\right)p\left({\delta}^2\right) $$

where *β*_*j*_ are the vectors of regression coefficients corresponding to the function *f*_*j*_. The full conditionals for the parameter vectors *β*_1_, …*β*_*p*_ as well as the full conditionals for *f*_*str*_, *f*_*ustr*_ and fixed effects parameters *γ*_*j*_ have known distributions. The inverse gamma distributions are used for these variance components *τ*^2^, *str*, *unstr* and (over all variance parameter *δ*^2^) [9, 18]. Thus, a Gibbs sampler can be used for MCMC simulation. BayesX 2.1 software version was used for the data analysis. Deviance Information Criterion (DIC) was used for model comparison.

## Results

### Exploratory Data Analysis

Table 1 indicates the distribution of childhood comorbidity and its related risk factors. The significance of the chi-square tests are indicated using the p-value. Results from cross-tabulation indicate that the sex of child, anemia level, breastfeeding, toilet facility place of delivery, family size, mother work status, mother educational level, childbirth order and source of drinking water are potential risk factors significantly associated with children comorbidity at 5% level of significance. For instance, the prevalence of having two illnesses is higher among the female children while having one disease among the male children (9.2, 15.3%, *p*-value = 0.011), respectively. The incidence of childhood comorbidity is high among anemic children than non-anemic children (*p*-value = 0.040). Besides, from Table 1, the place of delivery is highly associated with comorbidity (*p*-value < 0.000). The prevalence of having one or more illnesses is higher among children delivered at home than children delivered at a health center/hospital (15.8, 10.6 and 3.7%).

**Table 1:** Distribution of childhood comorbidity and its related selected risk factors

Covariates	Level	Comorbidity N (%)				*P*-value
		Had none of the three illnesses	Had one illness	Had two illnesses	Had three illnesses	
Sex of child	Male	3222 (72.3)	683 (15.3)	400 (9.0)	149 (3.3)	0.011
	Female	3173 (74.0)	579 (13.5)	394 (9.2)	142 (3.3)	
Anemia level	Anemic	6216 (73.0)	1236 (14.5)	779 (9.1)	289 (3.4)	0.040
	Non-anemic	179 (80.6)	26 (11.7)	15 (6.8)	2 (0.9)	
Breastfeeding	No	2114 (73.2)	432 (15.0)	261 (9.0)	80 (2.8)	0.035
	Yes	4281 (73.1)	830 (14.2)	533 (9.1)	211 (3.6)	
Type of toilet facility	flush toilet	250 (71.4)	52 (14.9)	37 (10.6)	11 (3.1)	0.050
	latrine	3253 (73.4)	669 (15.1)	380 (8.6)	130 (2.9)	
	no facility	2892 (73.0)	541 (13.7)	377 (9.5)	150 (3.8)	
Place of delivery	home	4231 (75.0)	771 (13.7)	465 (8.2)	176 (3.1)	0.000
	health center	2164 (69.8)	491 (15.8)	329 (10.6)	115 (3.7)	
Currently working	No	4680 (74.1)	891 (14.1)	552 (8.7)	189 (3.0)	0.002
	Yes	1715 (70.6)	371 (15.3)	242 (10.0)	102 (4.2)	
Household members	1–5 members	2747 (71.3)	610 (15.8)	361 (9.4)	136 (3.5)	0.009
	6–10 members	3457 (74.9)	607 (13.1)	407 (8.8)	146 (3.2)	
	above 10 members	191 (70.5)	45 (16.6)	26 (9.6)	9 (3.3)	
Highest educational level	No education	4160 (74.7)	730 (13.1)	495 (8.9)	181 (3.3)	0.000
	Primary	1578 (69.7)	370 (16.3)	230 (10.2)	86 (3.8)	
	Secondary and higher	657 (72.0)	162 (17.8)	69 (7.6)	24 (2.6)	
Birth order number	1st order	1203 (69.7)	303 (17.6)	160 (9.3)	60 (3.5)	0.002
	2-3rd order	2071 (73.8)	391 (13.9)	262 (9.3)	83 (3.0)	
	4th and above order	3121 (74.2)	568 (13.5)	372 (8.8)	148 (3.5)	
Source of drinking water	piped	848 (70.1)	204 (16.9)	122 (10.1)	36 (3.0)	0.038
	public tap	2943 (73.9)	549 (13.8)	366 (9.2)	123 (3.1)	
	protected spring	510 (73.7)	85 (12.3)	66 (9.5)	31 (4.5)	
	other	2094 (73.2)	424 (14.8)	240 (8.4)	101 (3.5)	

### Hotspot Analysis

**Fig. 2:**
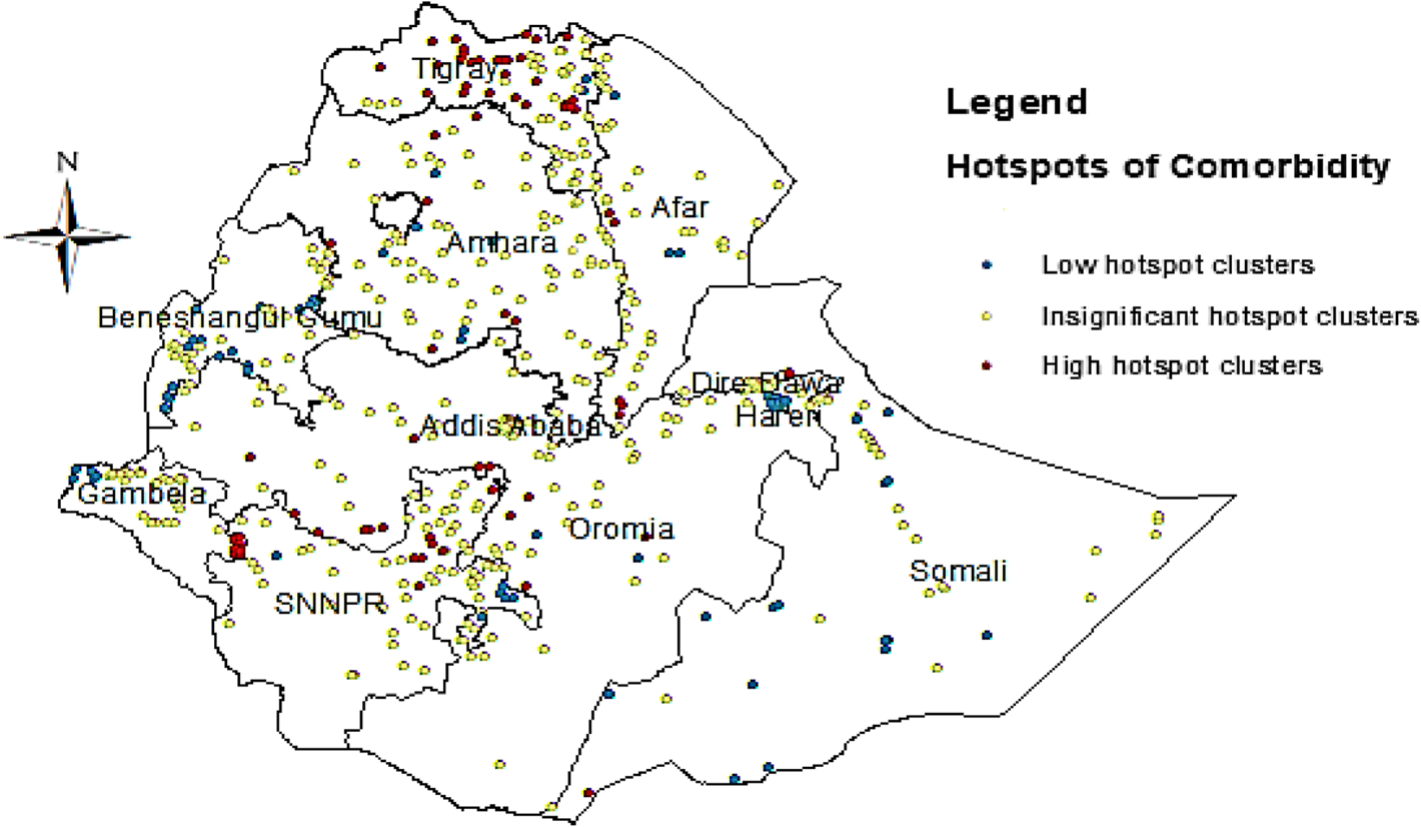
Hotspot and coldspot identification of childhood comorbidity at cluster level. ArcGIS 10.5 software version was used to plot the map

Figure 2 shows the spatial distribution of childhood comorbidity. Red colors indicate significant (*p*-value < 0.05) clusters of comorbidity areas, whereas, blue colors show significant coldspot areas. The regions of Tigray, Amhara, SNNPR, Gambella and Oromia were the hotspot regions in the country. Whereas, the regions of Harari, Benishangul-Gumuz and Somali were revealed as coldspot regions (Fig. 2 and Table 2).

**Table 2:** Hotspot and coldspot analysis of childhood comorbidity between clusters

Region	Overall clusters	High hotspots	Low hotspots	Insignificant hotspots
Tigray	63	29	2	32
Afar	53	5	3	45
Amhara	71	7	6	58
Oromia	74	13	2	59
Somali	67	1	14	52
Benishangul	50	–	17	37
SNNPR	71	7	6	58
Gambela	50	7	5	38
Harari	44	–	44	–
Addis Ababa	56	–	–	56
Dire Dawa	44	1	–	43

### Model fit

We fit and compare the following structured additive regression models to identify the best-fit model to the data and to examine factors related to the likelihood of comorbidity with diarrhea, fever and cough. The first model (M1) estimated fixed effects while the second model (M2) incorporates the nonlinear effect *f*(*child age*) without structured and unstructured effects. The third model (M3) considers the structured and unstructured effects and the fourth model (M4) added fixed effects to the third model. The fifth model (M5) includes fixed effects, nonlinear and structured effects. Finally, the sixth model (M6) includes both unstructured and structured effects besides linear and nonlinear effects.

9 $$ \mathrm{M}1:{\eta}_{ij k}={\beta}_0+{H}_{ij}{\beta}_k $$

10 $$ \mathrm{M}2:{\eta}_{ij k}={\beta}_0+{\mathrm{H}}_{ij}{\beta}_k+f(CAge) $$

11 $$ \mathrm{M}3:{\eta}_{ijk}={\beta}_0+{f}_{str}(region)+{f}_{unstr}(region) $$

12 $$ \mathrm{M}4:{\eta}_{ij k}={\beta}_0+{H}_{ij}{\beta}_k+{f}_{str}(region)+{f}_{unstr}(region) $$

13 $$ \mathrm{M}5:{\eta}_{ij k}={\beta}_0+{H}_{ij}{\beta}_k+f(CAge)+{f}_{str}(region) $$

14 $$ \mathrm{M}6:{\eta}_{ij k}={\beta}_0+{H}_{ij}{\beta}_k+f(CAge)+{f}_{str}(region)+{f}_{unstr}(region) $$

From Table 3, based on the DIC, the first model M1 has DIC = 14,594.047, while the second model M2 gave a DIC of 14,401.84 suggesting that the combined effects of linear and nonlinear explained the risk of comorbidity better than a fixed effect only. The third model M3 includes structured and unstructured effects only model which shows improvement than model M1. Furthermore, we considered linear, structured and unstructured effects (M4) with DIC = 14,428.595, which suggest the joint effects of linear, structured and unstructured, explained the risk of comorbidity better than M1 and M3. The fifth model M5 includes linear, nonlinear and spatial effect suggesting that the model best explain the risk of coexistence of diseases as compared to the other candidate models. The sixth model M6 includes both unstructured and structured effects besides linear and nonlinear effects. However, not shows improvement than M5. Hence, in this paper, we discuss the best model (M5).

**Table 3:** Comparison of model fit using the Deviance Information Criterion (DIC)

Model	Deviance	PD	DIC
M1	14,506.354	43.84641	14,594.047
M2	14,276.435	62.702432	14,401.84
M3	14,390.552	30.632699	14,451.817
M4	14,292.931	67.831814	14,428.595
M6	14,040.064	86.639755	14,213.344
M5	14,041.291	86.737705	14,214.766

### Fixed effects result from BGAMM

Table 4 provides the posterior mean estimates of fixed effects of the demographic, socioeconomic and environmental related risk factors of comorbidity using the multinomial logistic regression model. The result presented in the table indicates 95% credible intervals. Whereas, when the 95% credible intervals include zero, it shows the effect is not statistically significant at 5% level of significance. In addition, the table shows the estimated effects of categorical risk factors: child’s sex, anemia level, breastfeeding, types of toilet facility, mother’s work status, mother’s educational level, childbirth order and source of drinking water on the comorbidity of diarrhea, cough and fever in Ethiopia. From Table 4, the result indicated that female children were at decreased risk of illness relative to male children (OR: 0.84; 95% CI: 0.73, 0.95). Furthermore, from the same table, the odds of having combinations of the three illnesses (diarrhea, fever and cough) (OR: 0.23; 95% CI: 0.04, 0.85) were lower for non-anemic children relative to anemic children. Children breastfed were at decreased risk of comorbidity of diarrhea, fever and cough relative to non-breastfed children (OR: 0.77; 95% CI: 0.67, 1.11, OR: 0.79, 95% CI: 0.66, 0.95).

**Table 4:** Posterior mean estimates of fixed effect parameters for childhood comorbidity

Posterior odds ratio (CI: 95%)			
Factors	Child had only one illness	Child had two illnesses	Child had three illnesses
Constant	0.42 (0.22, 0.79)	0.15 (0.07, 0.32)	0.17 (0.03, 1.08)
Sex of child (Male = ref)			
Female	0.84 (0.73, 0.95)	0.97 (0.86, 1.16)	0.94 (0.76, 1.18)
Anemia (Anemic = ref)			
Non anemic	1.23 (0.54, 1.23)	0.76 (0.42, 1.27)	0.23 (0.04, 0.85)
Breast (Yes = No)			
Yes	0.77 (0.67, 0.89)	0.79 (0.65, 0.95)	0.89 (0.68, 1.18)
Types of toilet (No toilet = ref)			
Latrine	1.02 (0.73, 1.34)	0.77 (0.54, 1.13)	0.38 (0.18, 0.91)
Flush toilet	0.97 (0.68, 1.38)	0.92 (0.64, 1.39)	0.42 (0.18, 0.99)
Place of delivery (Health center = ref)			
Home	0.91 (0.77, 1.06)	1.15 (0.96, 1.38)	1.03 (0.76, 1.38)
Working staus (no = ref)			
Yes	1.13 (0.97, 1.29)	1.29 (1.09, 1.55)	1.66 (1.29, 2.20)
Household member (1–5)			
6–10 member	0.72 (0.53, 1.03)	0.96 (0.63, 1.40)	0.63 (0.37, 1.02)
Above 10 member	0.69 (3.25, 1.42)	1.19 (0.44, 2.75)	0.72 (0.14, 2.48)
Mother’s Education (No education = ref)			
Primary	0.84 (0.71, 0.79)	1.00 (0.83, 1.21)	1.15 (0.86, 1.58)
Secondary& higher	1.13 (0.89, 1.43)	0.56 (0.41, 0.78)	0.76 (0.45, 1.27)
Birth order (First order = ref)			
2-3rd order	0.79 (0.69, 0.94)	0.98 (0.79, 1.22)	0.98 (0.59, 1.65)
4th and above	0.81 (0.69, 0.95)	0.92 (0.74, 1.13)	0.95 (0.50, 1.84)
Source of drinking water (piped = ref)			
Public tap	0.99 (0.89, 1.13)	1.06 (0.92, 1.25)	0.94 (0.63, 1.40)
Protected spring	1.05 (0.89, 1.25)	1.27 (1.04, 1.49)	1.40 (0.89, 2.32)
Other	0.84 (0.65, 1.07)	0.76 (0.54, 1.04)	0.94 (0.63, 1.40)

Similarly, there were a significant relationships between latrine toilet and flush toilet (OR: 0.38; 95% CI: 0.18, 0.91) and (OR: 0.42; 95% CI: 0.18, 0.99) with multiple illnesses, respectively. This means that children from households use latrine toilets were at decreased risk of diarrhea, fever and cough illnesses compared to children from households with no toilet facilities. The result also indicated that children from households with flush toilet facilities were at low risk of diarrhea, fever and cough illnesses relative to children from households with no toilet facilities. The other important risk factor associated with childhood comorbidity was the mother’s current work status. Children from an employed mother were at high risk of comorbidity of diarrhea, fever and cough relative to children from unemployed mother (OR: 1.29; 95% CI: 1.09, 1.55) and (OR: 1.66; 95% CI: 1.29, 2.20).

Furthermore, the results indicated that children from secondary and higher educated mother were at reduced risk of two illnesses compared to children from uneducated mother (OR: 0.56, 95% CI: 0.41, 0.77). Additionally, childbirth order number is a significant risk factor for child illness. Children with birth order number higher were at decreased risk of comorbidity of diarrhea, fever and cough relative to the first birth order number. Household’s source of drinking water is associated to childhood illnesses. Children from households drink protected spring (OR: 1.27, 95% CI: 1.04, 1.55) were at high risk of two illnesses as compared to children from households drink piped water.

### Spatial effects

**Fig. 3:**
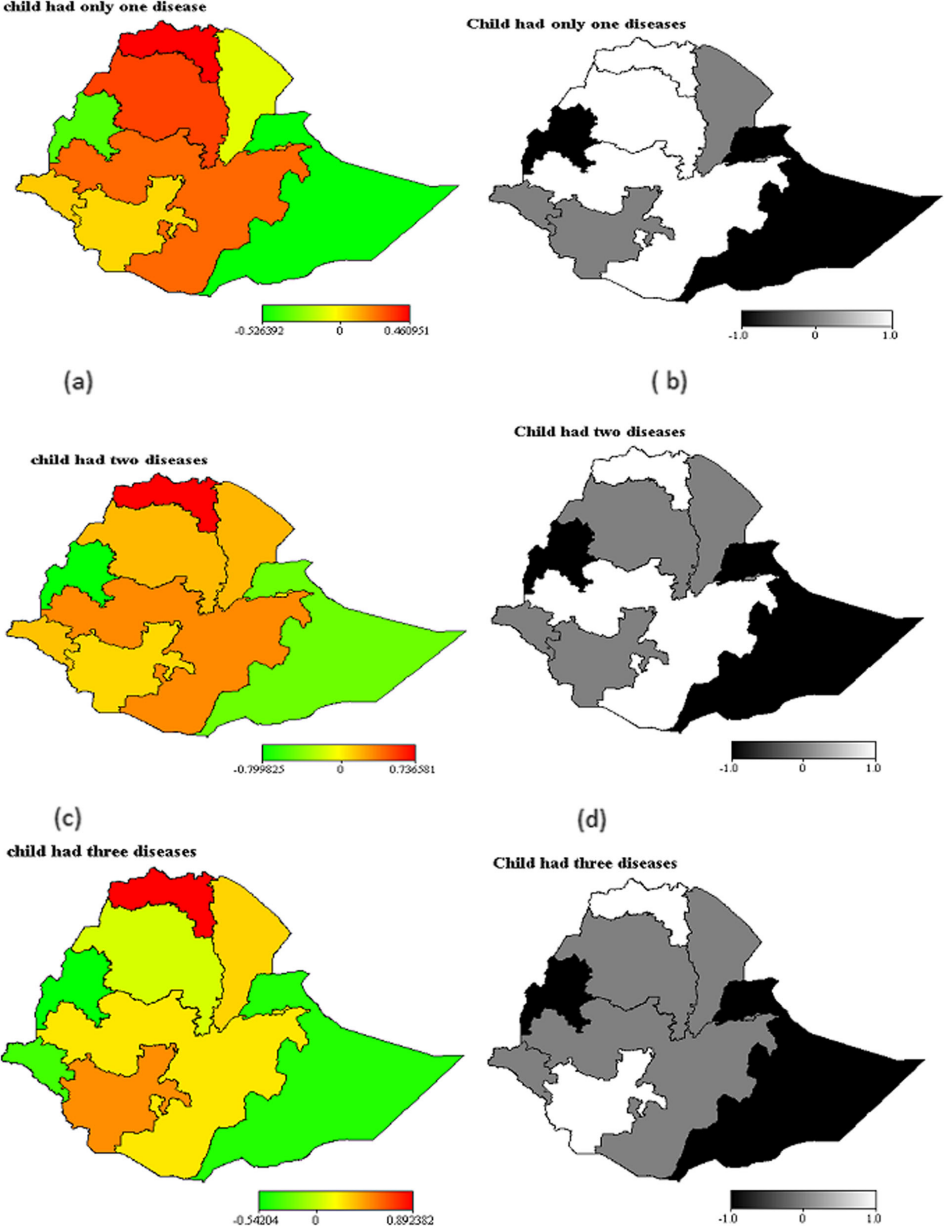
Estimated residual spatial region effects (left) and 95% posterior probability map of child comorbidities in Ethiopia (right). BayesX 2.1 software version was used to plot the maps. https://bayesx.software.informer.com/2.1/

Figure 3 (left) shows the multinomial estimated structured spatial effects of comorbidity. The result indicates a regional disparity in relative risk of a child exposed to a mixture of illnesses (diarrhea, fever and cough). From the map (left panel), the result shows the Northern and central regions of Ethiopia have high to medium prevalence (likelihood) of the only one illness. For instance, children residing in Tigray have the highest risk of having one of the diseases. Figure 3 (right panel) indicates the posterior probability maps of childhood comorbidity at a 95% credible interval. The regions in black shading are places where the estimates for specific illness were significantly lower, while the regions in white shading indicates places where the estimates for a specific illness were significantly higher. The regions in gray shading indicated an insignificant spatial effect on the child morbidity. Results from the maps show that children from Benishangul-Gumuz and the eastern part of the country were less likely to have suffered from comorbidity of diarrhea, fever and cough (b, d and f). Whereas, children lives in regions of Tigray, Amhara and Oromia were more likely to have suffered from one of the illnesses (a). Similarly, children from regions of Oromia and Tigray were more likely to at high risk of comorbidity of diarrhea, fever, or cough (d). Moreover, children from regions of Tigray and SNNPR have higher likelihood of suffering from three illnesses (f).

### Nonlinear effect results from BGAMM

**Fig. 4:**
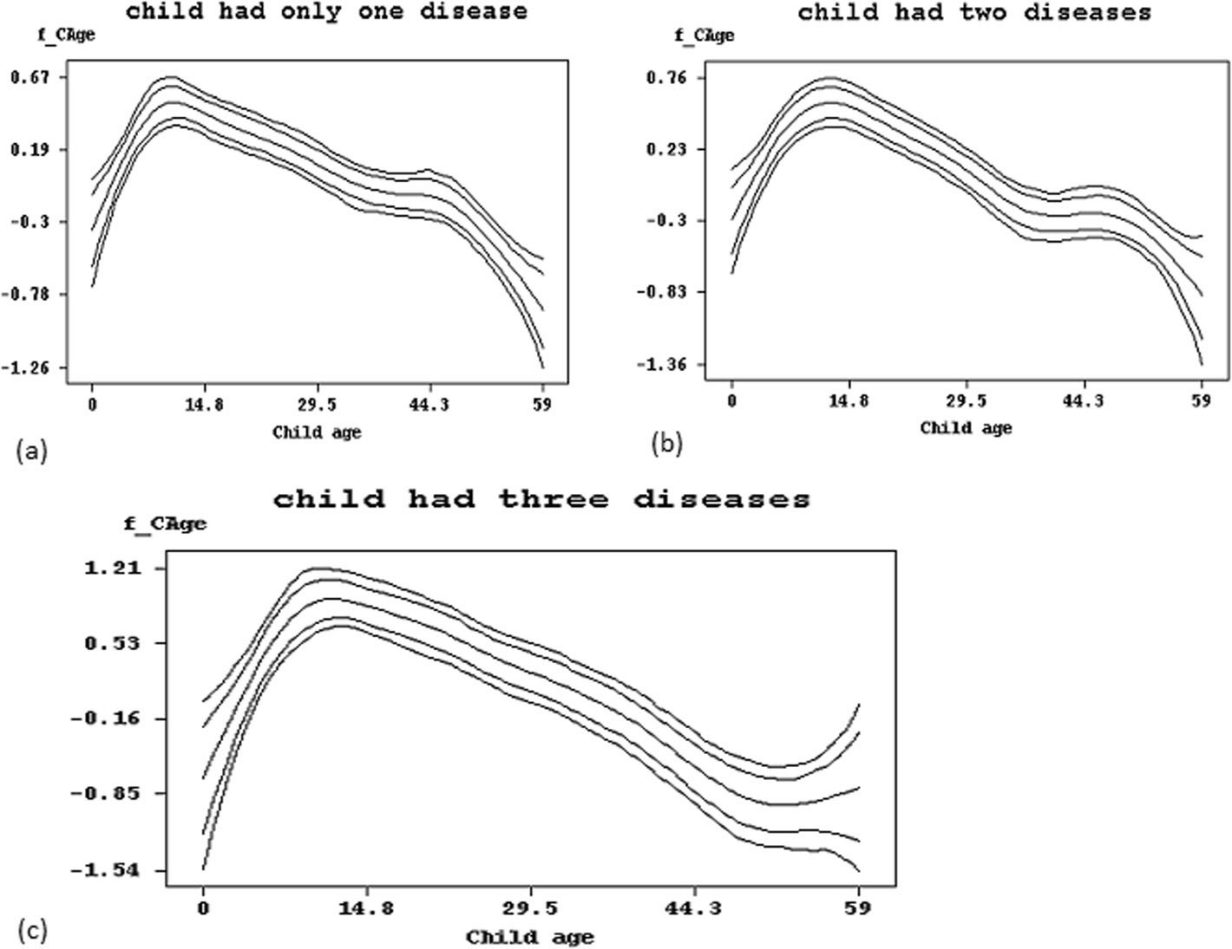
Nonlinear effects of child’s age (in months) on the comorbidity of diarrhea, fever and cough. BayesX 2.1 software version was used to plot the graphs. https://bayesx.software.informer.com/2.1/

The nonlinear effect components of the continuous covariate (child’s age in months) are portrayed in Fig. 4. It indicates the posterior model estimates of a child’s age together with 80% and 95% pointwise confidence intervals. The graphs are composed of five trend lines with the centerline is estimate of the posterior mean bounded by the two inner lines are 95% credible intervals and the outer lines are 80% credible intervals. Furthermore, the plots indicate a negative nonlinear effect of a child’s age on the probability of the illnesses and their coexistences. The results show that children suffer from any of cough, diarrhea, or fever as they grow older to zenith around aged 10 to 15 months and it becomes decreases as the child grows in age (Fig. 4 (a-c). The Figure for each type of co-morbidity indicates that the child’s health deteriorates quickly in 6–8 months. For instance, lower child age increases the risk of coexistence of diarrhea, fever and cough (Fig. 4 (c)).

## Discussion

This study focused on cross-sectional data analysis of childhood comorbidities in Ethiopia using an application of spatial multinomial logistic models. It is believed that an understanding of the dynamics of the co-occurrence of illnesses is vital in evaluating the health conditions of a community. The study revealed that sex of child, anemia level, breastfeeding, types of toilet facility, mother’s work status, source of drinking water, mother’s educational level and childbirth order have a significant effect on comorbidities of childhood diseases in Ethiopia.

The result of the sex of the child confirms with the findings of [5, 12] where the sex of child effect on childhood illnesses was statistically significant. The result suggested that male children were more affected by one of the illnesses than female children. This situation is attributed to sex discernment and biological causes in [12]. Likewise, the results reveal that the risk of the three illnesses (diarrhea, fever and cough) was higher among non- breastfed children than breastfed children. The result supports the findings of [6, 10, 19] as the not-breastfed children are in a worse relationship with infection. Moreover, the finding indicates that children from households that have no toilet facilities were associated with infection. Our finding is in agreement with previous studies [10, 12]. As a result, this study suggests that interventions on household sanitation reduce risks to which a child exposed.

Similarly, our findings showed that children with employed mothers were at high risk of having comorbidities. This is maybe due to the employed mother leave her child at home with the hands of caretakers. Because of this, the length of breastfeeding shortened and caretakers are usually uneducated [19, 20]. Also, the study found that the mother’s education level is an important risk factor affecting the wellbeing of children in the country. Children from lower educated mothers are more at high risk of one of the illnesses and two illnesses. The finding is consistent with previous studies [5, 21]. The other important focus of this study was to consider nonlinear effect components. The study result showed the negative nonlinear effect of the child’s age on the odds of comorbidities. Likewise, a high risk of one of the illnesses and illness combinations observed among children aged around 10–15 months. The result is in agreement with that of [6]. Of specific notice here is that the analysis found that there is a regional disparity in relative risk of the combination of comorbidity of diarrhea, fever and cough in Ethiopia. Tigray, Amhara, SNNPR, Gambella and Oromia were identified as hotspots regions while Harari, Benishangul-Gumuz and Somali were identified as coldspot regions of illnesses. This is might be due to different factors like socioeconomic, geographical, political and cultural differences among the regions. Residual risk estimates ranged from − 0.526 to 0.460 for a child who had only one illness, from − 0.799 to 0.736 for a child who had two illnesses and from − 0.542 to 0.893 for a child who had the illnesses comorbidities. Furthermore, the highest risk of the coexistence of cough, diarrhea and fever illnesses found in the Tigray region and SNNPR.

## Conclusions

Diarrhea, cough, and fever are the foremost causes of childhood diseases and mortality in Ethiopia. The central intention in this study was to specify areas of hotspot and coldspot risk of childhood comorbidity of diarrhea, fever and cough in Ethiopia. This investigation identified significant disparities in childhood illnesses across the regions of the country and the effect played by demographic, socioeconomic and environmental factors. The results offered the likelihood of simultaneously observing problems of compound illnesses in Ethiopia. Our analysis identified that regions of Amhara, Oromia, SNNPR and Tigray have a high risk of comorbidity and are more pretentious. In addition, the findings revealed that being male children, from a household’s lack of toilet facility, children from a family who use unsafe water, anemic children, children from working mothers, non-breastfed and children from uneducated mothers are at high risk of multiple illnesses. Younger children are at high risk of compound illness. Therefore, decision-makers should put more attention to targeting highly exposed regions as well as sociodemographic risk factors on childhood illness.

## Abbreviations

DHS: Demographic and Health survey
BGAMM: Bayesian Geoadditive Multinomial Model
